# Effects of medication management in geriatric patients who have fallen: results of the EMMA mixed-methods study

**DOI:** 10.1093/ageing/afae070

**Published:** 2024-04-15

**Authors:** Stephanie Clemens, Bernhard Iglseder, Reinhard Alzner, Magdalena Kogler, Olaf Rose, Patrick Kutschar, Simon Krutter, Karin Kanduth, Christina Dückelmann, Maria Flamm, Johanna Pachmayr

**Affiliations:** Institute of Pharmacy, Pharmaceutical Biology and Clinical Pharmacy, Paracelsus Medical University, Salzburg, Austria; Center of Public Health and Health Services Research, Paracelsus Medical University, Salzburg, Austria; Department of Geriatric Medicine, Christian Doppler Klinik, Paracelsus Medical University, Ignaz-Harrer-Straße 79, A-5020, Salzburg, Austria; Department of Geriatric Medicine, Christian Doppler Klinik, Paracelsus Medical University, Ignaz-Harrer-Straße 79, A-5020, Salzburg, Austria; Landesapotheke Salzburg, Salzburg, Austria; Institute of Pharmacy, Pharmaceutical Biology and Clinical Pharmacy, Paracelsus Medical University, Salzburg, Austria; Institute of Nursing Science and Practice, Paracelsus Medical University, Salzburg, Austria; Institute of Nursing Science and Practice, Paracelsus Medical University, Salzburg, Austria; Institute of Pharmacy, Pharmaceutical Biology and Clinical Pharmacy, Paracelsus Medical University, Salzburg, Austria; Institute of Pharmacy, Pharmaceutical Biology and Clinical Pharmacy, Paracelsus Medical University, Salzburg, Austria; Landesapotheke Salzburg, Salzburg, Austria; Institute of General Practice, Family Medicine and Preventive Medicine, Paracelsus Medical University, Salzburg, Austria; Institute of Pharmacy, Pharmaceutical Biology and Clinical Pharmacy, Paracelsus Medical University, Salzburg, Austria; Center of Public Health and Health Services Research, Paracelsus Medical University, Salzburg, Austria

**Keywords:** patient perspectives, medication management, fall-risk-increasing drugs, fracture, geriatrics, qualitative research, older people

## Abstract

**Background:**

comprehensive medication management (CMM) can reduce medication-related risks of falling. However, knowledge about inter-individual treatment effects and patient-related barriers remains scarce.

**Objective:**

to gain in-depth insights into how geriatric patients who have fallen view their medication-related risks of falling and to identify effects and barriers of a CMM in preventing falls.

**Design:**

complementary mixed-methods pre–post study, based on an embedded quasi-experimental model.

**Setting:**

geriatric fracture centre.

**Methods:**

qualitative, semi-structured interviews framed the CMM intervention, including a follow-up period of 12 weeks. Interviews explored themes of falling, medication-related risks, post-discharge acceptability and sustainability of interventions using qualitative content analysis. Optimisation of pharmacotherapy was assessed via changes in the weighted and summated Medication Appropriateness Index (MAI) score, number of fall-risk-increasing drugs (FRID) and potentially inappropriate medications (PIM) according to the Fit fOR The Aged and PRISCUS lists using parametric testing.

**Results:**

thirty community-dwelling patients aged ≥65 years, taking ≥5 drugs and admitted after an injurious fall were recruited. The MAI was significantly reduced, but number of FRID and PIM remained largely unchanged. Many patients were open to medication reduction/discontinuation, but expressed fear when it came to their personal medication. Psychosocial issues and pain increased the number of indications. Safe alternatives for FRID were frequently not available. Psychosocial burden of living alone, fear, lack of supportive care and insomnia increased after discharge.

**Conclusion:**

as patients’ individual attitudes towards trauma and medication were not predictable, an individual and longitudinal CMM is required. A standardised approach is not helpful in this population.

## Graphical Abstract

**Graphical Abstract:**
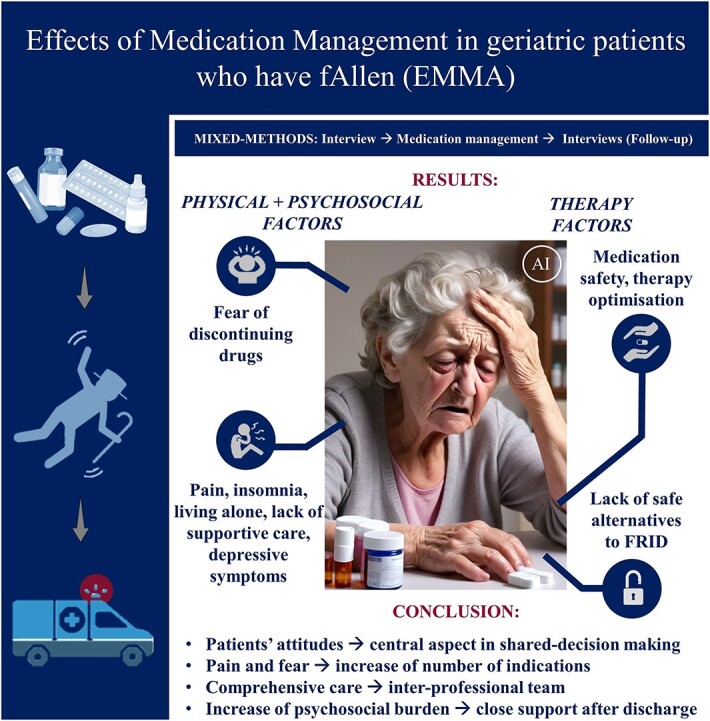

(AI: created with Artificial Intelligence).

## Key points

- While most patients agreed to a reduction in pill burden as a general goal, their opinion changed when it came to their own medication.
- Fear and pain are major burdens after fractures and needed to be addressed with specific drugs.
- Safer alternatives to fall-risk-increasing drugs were hardly available.
- Pharmacists’, geriatricians’ and orthopaedists’/traumatologists’ foci clearly differed, which indicates the need of inter-professional collaboration.
- As psychosocial issues emerged after discharge and medication frequently changed, a need for close support was seen during this most vulnerable phase.

## Introduction

Polypharmacy and falls in geriatric patients remain a tremendous global health problem [1–3]. The Global Burden of Disease study in 2017 reported nearly 36 million disability-adjusted life years, resulting from falls [1].

Pharmacotherapy in older patients requires a thoughtful balance between optimising therapy and minimising medication risks [4, 5]. Comprehensive medication management (CMM) defines a medication review process with a collaborative approach, to assess patients’ drug regimen and optimise drug therapy [6]. CMM can be an important intervention for a safer and more effective use of drugs [7, 8].

There is growing awareness that the optimisation of pharmacotherapy in geriatric patients who have fallen is not limited to discontinuing drugs in extensive polypharmacy. Contrariwise, under-treatment is widespread amongst these patients [9]. A scoping review by Dabkowski *et al*. demonstrated the importance of partnering with patients [10]. A therapeutic challenge is the multifactorial nature of falls and the required specific expertise in prevention and care of multimorbid patients [11–13]. Results of CMM in preventing falls vary widely between available studies [14–18]. Holistic approaches for practical, safe and sustainable care of high-risk patients were suggested by the Global Falls Guideline Task Force [19, 20]. Hospital discharge and transition to the community setting represent a vulnerable phase in the care pathway of geriatric patients [21]. Research on approaches on fall prevention implementation and patient experiences of CMM to evaluate the intervention are scarce [13, 19, 22].

Mixed-methods research is helpful to map a comprehensive picture of patient-centred interventions through qualitative (in-depth perceptions) and quantitative (interventional) findings [23]. This mixed-methods study examined the implementation of a CMM process to provide better insights into individual perspectives of geriatric patients who have fallen on the medication-related risk of falling, on identifying organisational and medico-psychosocial effects as well as on implementation challenges in a geriatric fracture setting.

## Methods

Details of the study methodology and study protocol have been published previously and are summarised in the present manuscript [24]. Research questions were built on basis of the PICOS framework [25]. The Medical Research Council (MRC) guidance served as an overall framework on developing and evaluating the complex intervention of CMM focusing on fall-risk reduction [26].

### Design

The study design was a prospective, monocentric, single-arm, longitudinal mixed-methods pre–post-interventional study, using a complementary approach based on an embedded quasi-experimental model according to Creswell [27]. The study design focuses on exploring the process of a real-world practice fall prevention intervention and on a follow-up on the results [27].

Qualitative, semi-structured interviews with patients framed the CMM intervention, including a follow-up period of 12 weeks. Pre-interventional face-to-face interviews were arranged upon patients’ hospital admission (T1). Post-interventional interviews were conducted remotely after discharge at three points of time (2, 6 and 12 weeks) via telephone (T5, T6, T7). Pre-interventional interviews included four themes: (1) fall experience/fall event and relation to medication; (2) knowledge on the indication of the medication; (3) prescriptions on polymedication and discontinuing drugs; (4) therapy adherence. Post-interventional interviews comprised three themes: (1) health condition and reoccurrence of falling; (2) medication status; (3) satisfaction with the CMM service. Pre-tests of both interview guides (pre- and post-interventional) were performed within true patient involvement.

### Recruitment and data collection

Patients were recruited by the study geriatrician at a certified geriatric fracture centre (GFC) of a tertiary-care university hospital in Austria [28]. Recruitment was based on criterion sampling, reaching a diverse sample with respect to demographic characteristics, medication changes, fall experiences and the living situation. Data were collected from geriatric patients who have fallen between May 2021 and June 2023. All interviews were conducted by a trained researcher. Medication and health related data were collected via patients’ medical hospital charts. Participants’ inclusion criteria were ≥65 years old, community dwelling or living in assisted or independent living communities, on ≥5 long-term medications, hospitalised after an injurious fall and German speaking. All participants needed to be able and willing to take part in the intervention and needed to show the mental capacity (with a predicted Mini-Mental State Examination cut-off of >25/30) to give informed consent. The target sample size using G*Power calculation was 30 patients (for a paired *t*-test), based on 80% power (test *α* level of 0.05) to reach a medium effect size (*d* = 0.46) due to the use of the intervention (Appendix 1) [29].

### Intervention

The CMM intervention was based on the model of Collaborative Pharmacy Practice of the International Pharmaceutical Federation [30] and on standards in medication review by Rose *et al*. [31]. Core elements of the intervention were further inspired by the American Pharmacists Association and National Association of Chain Drug Stores Foundation [32]. The intervention consisted of five steps: (a) recording medication at admission by physicians, (b) a comprehensive medication review (Pharmaceutical Care Network, PCNE type 3) by a pharmacist [33], (c) discussion between a geriatrician and a pharmacist on pharmacotherapy optimisation, (d) communication with patients (shared decision-making) and (e) documentation of the optimised medication in the patient’s discharge letter by orthopaedists/traumatologists.

The CMM implementation included inter-professional engagement of study authors involved in the planning and measurement of the caring processes following the MRC. Programme outcomes were characterised according to a combined model of the Consolidated Framework for Implementation Research and a study by Proctor *et al*. [34, 35].

### Variables, instruments and outcomes

Patient-reported outcomes (primary outcomes) were assessed qualitatively by the use of guided, semi-structured interviews. Clinical and organisational outcomes (secondary outcomes) were measured quantitatively through pre–post-interventional changes in the

(a) Weighted and summated Medication Appropriateness Index score (MAI), assessed by two independent researchers [36],
(b) Number of fall-risk-increasing drugs (FRID), list compiled by a focused literature review [37–43]:

1. Psychotropic drugs (benzodiazepines, hypnotics, antipsychotics, antidepressants, antiepileptic medication)
2. Cardiovascular drugs (beta-blockers, alpha-blockers, diuretics, calcium channel blockers, angiotensin-converting enzyme inhibitors, angiotensin-II receptor antagonists, antiarrhythmics)
3. Antidiabetic drugs (insulins, sulfonylureas)
4. Anticholinergic drugs (antimuscarinics, skeletal muscle relaxants)
5. Analgesics (opioids, NSAR)
6. Eye drops

(c) Number of potentially inappropriate medications (PIMs), rated by the Fit fOR The Aged (FORTA) [44] and PRISCUS lists [45],
(d) Use of long-term medication (>4 weeks) as opposed by short-term medication, defined as a prescription for a short-term illness or condition expected to resolve within 30 days,
(e) Pill burden (total number of doses per day).

To explore the pre–post FRID exposure, FRID were sub-grouped to FORTA classes A–D (evidence) [44] and Anatomical Therapeutic Chemical Classification codes (therapeutic classes) [46].

### Analysis

**Figure 1:**
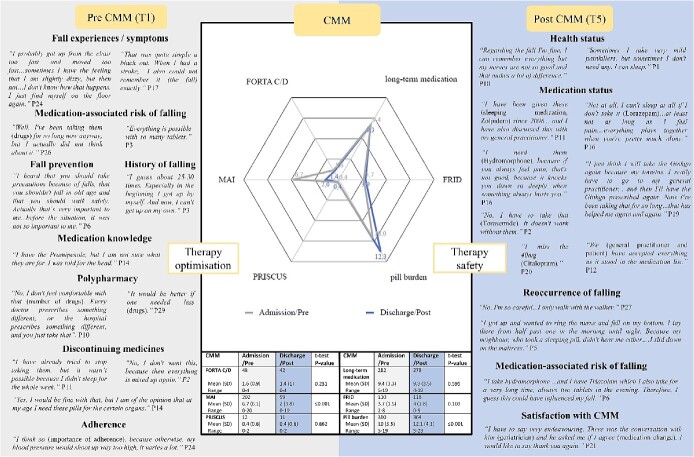
Mixed-methods matrix of the comprehensive medication management (CMM) outcomes (web chart) embedded in patients’ pre-interventional (grey area) and post-interventional (blue area) perceptions [36, 44, 45]. Patients’ most representative statements are displayed amongst categories. The web chart represents the medication status at admission (grey line) versus at discharge (blue line). Units are expressed as absolute numbers, means, SD, ranges and *P-*values. MAI, weighted and summated Medication Appropriateness Index; FRID, fall-risk-increasing drugs; PIM, potentially inappropriate medications; FORTA, Fit fOR The Aged list.

Interviews were audiotaped, transcribed verbatim and pseudonymised. Data were analysed by qualitative content analysis according to Mayring, using the technique of structuration with a combination of inductive category development and deductive category application [47]. The qualitative software MAXQDA 2022 (VERBI Software GmbH, Germany) was used to assist the analysis. The Standards for Reporting Qualitative Research were applied for reporting the qualitative outcomes of the EMMA study (Appendix 2) [48]. Quantitative data were analysed by descriptive statistics and parametric testing using IBM SPSS 27 (IBM, Chicago, IL, USA). Paired *t*-tests were performed to compare pre–post-interventional situations of the weighted and summated MAI, number of PIM, FRID, use of long-term medication and pill burden. Participants who missed a follow-up phone call remained in the analyses to retrieve a maximum of qualitative information. Corresponding quantitative data (e.g. number of reoccurrence of falls) of nonresponding participants were included in the final analysis via intention-to-treat analysis, following multiple imputation [49]. To complementary integrate qualitative and quantitative findings of the study, visual joint displays were used as a mixed-methods matrix [50] (Figure 1). Credibility, transferability, dependability and confirmability were key quality indicators to ensure rigour of the mixed-methods approach [51].

### Ethical considerations

The study has received ethical approval from the local ethics committee of Salzburg County, Austria (ID: 1059/2021) and was registered in the German Clinical Trials Register/Deutsches Register Klinischer Studien (ID: DRKS00026739). All study participants provided written informed consent.

## Results

### Recruitment, completion rates and missing data

Thirty geriatric patients who have fallen were recruited. All patients participated and completed the pre-interventional interviews and the five-step interventional CMM process (quantitative data). In total, 23/30 patients (76.6%) completed the full course of the study. Reasons for the loss to follow-up were a withdrawal of consent and a new-onset delirium (2/30, 6.7%). Nonresponse led to partial missing data in five participants (5/30, 16.6%) (Appendix 3).

### Study participants

Participants had various types of fractures and mostly lived alone. Details of the study participants are displayed in Table 1.

**Table 1:** Characteristics of study participants. Pre-interventional perspectives

**Patient characteristics**	**Total *n* = 30 (%)**
**Age, years**	
Mean (SD)	82.4 (5.1)
Range	72–93
**Sex**	
Female	22 (73.3)
Male	8 (26.6)
**Educational level**	
Primary education (or less)	6 (20)
Secondary education	23 (76.7)
Tertiary education (or higher)	1 (3.3)
**Living situation**	
Living alone	21 (70)
Living as a couple	7 (23.3)
Others (with daughter; grandchild)	2 (6.7)
**Caring situation**	
Partner	2 (6.7)
Family/relatives (only)	13 (43.3)
Family/relatives + help by social care; cleaning; mobile nursing care	5 (16.7)
No care	3 (10)
Others (home care service; help with cooking and cleaning)	7 (23.3)
**Cognitive performance (MMSE**)	
Mean (SD)	27.3 (0.9)
Range	26–28
**Charlson Comorbidity Index**	
Mean (SD)	3.1 (2.3)
Range	0–9
**Number of drugs at admission**	
Mean (SD)	10 (3.5)
Range	5–19
**History of falls**	
Previous falls (up to 30 repetitive reported falls)	25 (83.3)
First fall event	5 (16.6)
**Circumstances of the fall (activities during pre-fall phase)**	
Balance challenging (e.g. walking on stairs)	8 (26.6)
Positional change (sit-to-stand/walk)	7 (23.3)
Standing (standing still, standing while banding/reaching)	5 (16.6)
Walking (forward walking, turning while walking)	10 (33.3)
**Fractures and injuries (≥1 per patient)**	
Arm/hand (wrist fracture, upper arm fracture)	5 (16.7)
Head/face (skull-base fracture, skull/brain injury, head contusion, nasal bone fracture)	6 (20)
Hip/femur (femur shaft fracture, hip fracture, hip dislocation)	9 (28.1)
Rip fracture	2 (6.7)
Spine (spine fracture, sacral fracture)	7 (23.3)
Other (knee injury, long lie trauma)	2 (6.7)
**Length of stay in the study unit, days**	
Median (IQR)	9.5 (6.5)
Range	2–48

Units are expressed as number (percentage) unless otherwise stated.

^*^SD, standard deviation; IQR, interquartile range; MMSE, Mini-Mental State Examination

### Symptoms before the fall and relation to medication

Reported symptoms were grouped into three main categories [52]:

- Physiological symptoms of dizziness, light-headedness and drowsiness, including reported inattention (50%, 15/30),
- Reduced physiological function and loss of balance, including gait unsteadiness and stumbling (26.6%, 8/30) and
- Unexplained falls, including blackout, fainting and unconsciousness (23.3%, 7/30).

Most participants expected no relation between drugs and the events of falling, as they used the drugs for a long time and had strong beliefs in their benefits.

### Medication knowledge

Almost half of the participants (46.6%, 14/30) reported uncertainties relating to their drugs. Many participants felt excellently informed about their drugs but could not name them, nor their indication. Reasons were the high number of drugs, frequent switching of drugs and the general feeling of being overwhelmed. One participant reported:

‘*How I broke down when my wife died… it happened so quickly, really quickly… since then, my brain no longer functions.*’ P3.

### Reduction/discontinuing drugs

Many patients were initially open for amendments on their medication (56.6%, 17/30). The strongest reason was the excessive number of prescribed drugs due to hospitalisations and physicians’ visits. Nevertheless, almost half of the patients (43.3%, 13/30) ultimately rejected any discontinuation or dose reduction of their medication. Reasons were previous negative experiences with stopping a medication, resignation due to lack of prescriber communication caused by time issues, induced stress, strong beliefs in the need of medication (feeling better, healthier, enabling to be active) and high trust in physicians and their decisions. When it came to possible medication optimisations, most patients expressed fear and doubt. One patient stated:

‘*I don’t know how my health will react. My condition changes, sometimes I feel better, sometimes not so well.*’ P8.

### CMM intervention

**Figure 2:**
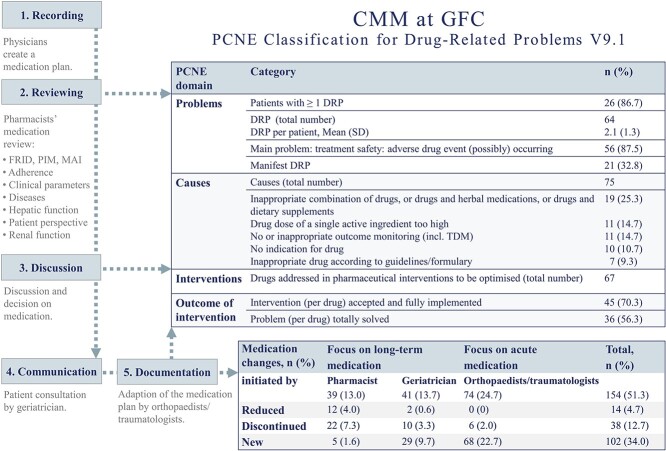
Comprehensive medication management (CMM) intervention: Pharmaceutical Care Network Europe (PCNE) classification for drug-related problems (DRP) [53] in accordance with the 5-step interventional CMM process. Only most relevant PCNE categories are listed. Medication change rate was calculated on basis of number of drugs at admission (pill burden; *n* = 300 drugs). Units are expressed as number (percentage) unless otherwise stated. SD, standard deviation; GFC, geriatric fracture centre; TDM, therapeutic drug monitoring.

Due to participants’ medication review, 64 drug-related problems (DRPs) were documented, with a mean (SD) of 2.1 (1.3) DRP for each patient (Figure 2). The main problems (87.5%, 56/64) were related to treatment safety issues with potential adverse drug events occurring. The medical acceptance rate of pharmaceutical suggestions was 70.3% (45/67). At patients’ discharge, 56.3% (36/67) of problems per drug were totally solved. Drug changes initiated by pharmacists and physicians were categorised as reduced doses, discontinued drugs or newly prescribed drugs. Pharmacists and geriatricians mainly focused on long-term medication, whereas orthopaedists/traumatologists managed acute medication. Medication discontinuation (most present: diuretics) was frequently initiated by pharmacists while orthopaedists/traumatologists (most present: analgesics) and the geriatrician (most present: vitamin D, calcium, denosumab) prescribed new drugs related to pain and osteoporotic management, respectively (Appendix 4).

### Therapy optimisation and therapy safety

On basis of the CMM, participants’ weighted and summated MAI score decreased significantly from a mean of 6.7 (SD: 6.1) to 2 (SD: 3.8, *P* ≤ 0.001) per patient.

This was in contrast to a significant increase of pill burden from a mean 10 (SD: 3.5) to 12.1 (SD: 4.1) drugs per patient (*P* ≤ 0.001). Reasons were postoperative indications for analgesics, antibiotics and antithrombotics. Although new drugs have been started, the number of prescribed FRID remained largely unchanged (*P* = 0.103; ≤4 FRID per patient).

Participants’ PIM, identified via the PRISCUS list, persisted low and remained largely unchanged (*P* = 0.662) as did PIM regarding FORTA classes C and D (≤1.6 prescriptions per patient) (Appendix 5). Pre–post changes (*P* = 0.595) in participants’ long-term medication (≤9.4 drugs per patient) were marginal.

### Post-interventional perspectives

#### Health condition and medication status

While two-thirds of participants reported good, very good and excellent self-rated health conditions, one-third stated a decreased health condition state over the time of 12 weeks post-discharge. Almost half of the participants experienced post-discharge medication changes (mainly antidepressants, sedatives, benzodiazepines, diuretics, analgesics and antihypertensives). Some participants reported not getting along with stopped/reduced drugs (especially antidepressants, sedatives, benzodiazepines), as the need for these drugs increased. Participants were exposed to several post-discharge physiological issues comprising sleep problems, fatigue, immobility (inability to perform daily activities), dizziness, pain, blood pressure fluctuations and blood sugar imbalances. One patient stated:

‘*Yesterday, I got such high blood pressure; I went to the general practitioner again. With 88 years, one becomes quite confused because one time I have to take these—the other time those drugs.*’ P10; T6.

#### Reoccurrence of falling and relation to medication

During the follow-up period, five participants experienced a recurrent fall at one of the three points in time. Falls were mainly caused by dizziness or were unexplained falls. In some cases, they were due to the similar pre-interventional causes of falling. Some falls were reported as in-hospital falls (out of bed, premature ward-leave) and accidental falls (car accident, e-scooter and walker).

#### Satisfaction with CMM

Participants expressed satisfaction with the CMM and appreciated the integration into decision-making for medication optimisation. Participants particularly mentioned shortage of staff and the wish to get more information from nurses when receiving their daily drugs. One patient expressed:

‘*They don’t have the staff; there is nothing you can do about it.*’ P30; T5.

#### CMM implementation outcomes

**Figure 3:**
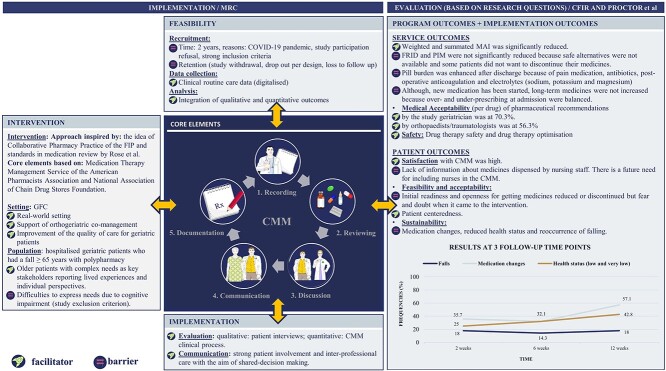
Development and evaluation of the complex CMM fall prevention intervention [26, 30–32, 34, 35] (created with Servier Medical Art) [54]. The core elements of the CMM are surrounded by characteristics of the circumstances (rated as facilitators/barriers) under which the intervention was designed, developed, implemented and evaluated. Programme and implementation outcomes are based on the qualitative and quantitative study results. CMM, comprehensive medication management; MRC, Medical Research Council; CFIR, Consolidated Framework for Implementation Research; GFC, geriatric fracture centre; FIP, International Pharmaceutical Federation; MAI, weighted and summated Medication Appropriateness Index; FRID, fall-risk-increasing drugs; PIM, potentially inappropriate medications.

Patient-centredness, shared decision-making, therapy safety and high patient satisfaction strengthened the CMM implementation. Barriers were patients’ fears and doubts of discontinuing drugs and limited sustainability, caused by increasing post-discharge medication changes, reducing self-rated health status and reoccurrence of falling (Figure 3).

### Facilitators and barriers to FRID reduction

**Figure 4:**
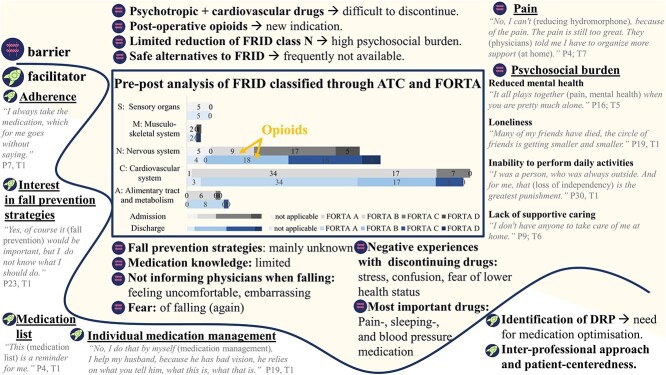
Facilitators and barriers for reducing fall-risk-increasing drugs (FRID) by a CMM intervention (mixed-methods matrix) [44, 46]. The bar chart depicts the absolute number of FRID of the study participants (*n* = 30) at admission (grey bar) versus at discharge (blue bar). The analysis is framed by patients’ most representative statements and summarised conclusions rated as facilitators (white area) or barriers (yellow area) of FRID reduction. ATC, Anatomical Therapeutic Chemical Classification; FORTA, Fit fOR The Aged list; DRP, drug-related problem.

Key facilitators for reducing FRID were patients’ close adherence, self-contained medication management by using medication lists and strong engagement. Nevertheless, the medical–psychosocial burden was identified as a considerable challenge to implementation feasibility, acceptability and sustainability (Figure 4):

- Medical issues (comorbidity, polypharmacy, FRID exposure, postoperative pain management)
- Mental issues (frustration, fear of falling, impatience with therapy goals, admission to nursing homes, desperation, depressive symptoms, doubt)
- Social issues (living alone, loss of independency, overwhelming caring situations of the partner, lack of supportive caring, loss of partner and friends)

A barrier to FRID reduction was newly initiated pain control medication due to the fracture event. The most frequently noticed categories of FRID were related to the ‘cardiovascular system’ and the ‘nervous system’. Noticeably, most of the FRID were grouped into FORTA classes A (indispensable) and B (beneficial). While the number of FRID of the cardiovascular system largely remained unchanged, the number of FRID of the nervous system increased. Reasons were frequent lacks of save alternatives to FRID, participants’ indications for sedatives/antidepressants and postoperative pain management (prescriptions of opioids).

## Discussion

The EMMA study explored the perspectives of geriatric patients who have fallen on CMM by a mixed-methods approach. It was found that the CMM improved medication quality, as shown by a significant decrease in the MAI. This result is noteworthy, as on the other hand the number of drugs including FRID increased due to surgery and hospitalisation (post-traumatic pain control, emotional distress).

The qualitative part of the study led to deeper insights into patients’ perceptions. Patients suspected no association between their drugs and falls. They strongly believed in the benefits but showed limited knowledge about their own medication. Patients uttered concerns towards discontinuation of almost any drug. In particular, there was a strong disparity between the agreement to discontinue drugs in general and the denial to reduce any drug of the own medication. Patients’ perceptions were included into all recommendations on medication optimisations (shared decision-making). A main trigger was patients’ strong belief in their medication at the time of the intervention, which was a delicate post-fracture situation. This finding is in line with previous studies, which found major health events to be strong barriers for drug discontinuation, but also highlighted low health literacy in this context [55–57]. However, attitudes towards medication can change over time, with the setting and depending on the counselling healthcare provider. This finding is most likely not limited to geriatric patients who have fallen. Weir *et al*. identified different personalities of patients: attitudes varied from high trust in medication to not giving much thought about it. The study consequently emphasised the necessity of an individual approach to older patients [58].

Another barrier to reduce pill burden was that fracture itself led to an increase of indications. Pain was the major symptom leading to the prescription of analgesics (mainly opioids), which is supported by guidelines for postoperative bone healing [59, 60] and by FORTA B (beneficial). Saver alternatives to this FRID class are not available. Consequently, analgesic FRID should not be regarded as a disadvantageous drug therapy in this scenario but should rather be seen in context of the patients’ needs and symptoms. Especially in complex geriatric patients, evidence-based and real-life orientated lists (e.g. as the FORTA list) might help to balance benefits and risks [61].

Within the inter-professional CMM, pharmacists’ and physicians’ foci clearly differed. While the pharmacists focused on optimising long-term medication including reduction of FRID and PIM, the geriatricians had an emphasis on osteoporosis management. Orthopaedists/traumatologists rather tackled acute medication, mainly related to pain management. Study patients appreciated detailed drug information within the CMM. Interdependencies between different roles, scopes and expertise are a core competence of team collaboration and enhance the delivery of patient-centred care [62].

Another finding of the study is that CMM showed a limited longitudinal effect after discharge. Half of the participants experienced multiple changes in their (self-rated) health situation. Medication was adapted in multiple ways and falls reoccurred during the follow-up period. A main burden of the participants were again psychosocial factors, namely living alone, sleeping issues and lack of participation in activities of daily life, which intensified over time. Psychosocial aspects of e.g. perceived loneliness and of symptoms of depression are known to have a strong impact on satisfaction with life [63]. Consequently, antidepressants, sedatives and benzodiazepines were the most frequent drug classes restarted after discharge. Walsh *et al*. and Boyé *et al*. described similar relapses to a formerly problematic medication after discharge [64, 65]. The observed increase in mental illness after fractures surely leads to new medication-related problems. A lesson learned from the study is that key factors for sustainability of CMM include accurate patient information on medication and strengthening the inter-professional communication between hospital and primary care. This could be reached through detailed information on medication optimisation in hospital discharge letters. Higher sustainability can also be reached by shifting the focus towards the needs of older patients and routinely incorporate deprescribing, as recommended by the position paper of the European Geriatric Medicine Society [66].

### Strengths and limitations

Notable strengths of the EMMA study were the (a) pre–post quasi-experimental design; (b) inclusion of community-dwelling geriatric patients who have fallen varying in age, sex, level of comorbidities and medication; (c) collaborative, inter-professional and patient-centred approach; (d) assessment of CMM implementation measures in a real-world setting, using the well-established MRC framework.

Despite these strengths, the study has several limitations. The EMMA study was conducted at only one GFC, without controls and with a small sample size, leading to limited generalisability. Patients with cognitive impairment were excluded. The recruiting geriatrician, the interviewing pharmacist and the fracture event may have influenced participants’ statements. Under- or, vice versa, over-reporting of experiences (recall bias) could have affected category classification. Culture and European ethnicity may influence how falling and CMM is experienced and limit transferability. However, it is not expected that this GFC and its patients differ substantially from other GFCs.

## Conclusion

The EMMA study adds new and detailed insights of CMM effects during hospitalisation and after transition of care. While quality of medication increased due to the CMM, the fracture event led to initiation of several new drugs, mainly analgesics and many of them listed as FRID. The perspective on medication clearly differed between patients, pharmacists, geriatricians and orthopaedists/traumatologists. Medication frequently changed after discharge, leading to a limited longitudinal effect of the CMM. As a conclusion, a CMM in hospital should take the different perspectives into account. Clinical reasoning is a central part. Patients require tailored and individual CMM, addressing their beliefs and expectations. CMM based on a mere data analysis and without discussing patients’ needs and problems does not seem to be promising. Hence, shared decision-making and addressing chief complaints should be an integral part of any CMM. As medication is prone to change due to psychosocial aspects shortly after discharge, CMM should be provided in a repetitive or longitudinal manner. More research on implementation aspects of inter-professional CMM in different settings is requested to explore different facets of personalities.

## Supplementary Material

aa-23-1793-File007_afae070

## Data Availability

Research data are not shared due to privacy and ethical restrictions.
